# Audiometric threshold shifts after total knee arthroplasty by using gentamicin-loaded bone cement

**DOI:** 10.3906/sag-1710-135

**Published:** 2019-04-18

**Authors:** Adem ÇÖBDEN, Yalkın ÇAMURCU, Serap BULUT ÇÖBDEN, Hakan SOFU, Hanifi ÜÇPUNAR, Ahmet SEVENCAN, Hüseyin DEMİREL

**Affiliations:** 1 Department of Orthopedics and Traumatology, Ministry of Health, Kayseri City Hospital, Kayseri Turkey; 2 Department of Orthopedics and Traumatology, Faculty of Medicine, Erzincan Binali Yıldırım University, Erzincan Turkey; 3 Department of Otorhinolaryngology, Ministry of Health, Kayseri City Hospital, Kayseri Turkey; 4 Department of Orthopedics and Traumatology, Bahçelievler Medical Park Hospital, İstanbul Turkey; 5 Department of Orthopedics and Traumatology, Şanlıurfa Education and Research Hospital, Şanlıurfa Turkey; 6 Department of Orthopedics and Traumatology, Sivas Numune Hospital, Sivas Turkey

**Keywords:** Gentamicin, ototoxicity, total knee arthroplasty, antibiotic-loaded bone cement, audiometry

## Abstract

**Background/aim:**

The aim of this study was to investigate postoperative audiometric threshold shifts in patients who underwent primary total knee arthroplasty (TKA) using gentamicin-loaded bone cement (GLBC) in comparison with the ones who underwent TKA without GLBC.

**Materials and methods:**

Forty patients (gentamicin group) who underwent primary TKA using GLBC and 29 patients (control group) who underwent primary TKA using standard bone cement were included in this prospective case-control study. Baseline pure-tone audiometric evaluation was performed preoperatively and repeated at the postoperative third day for all patients. Control audiometric evaluation was performed weekly for patients who were diagnosed with ototoxicity according to audiometric threshold shifts.

**Results:**

Ototoxicity was diagnosed in 8 of 40 patients (20%) in the gentamicin group according to postoperative audiometric threshold shifts, whereas no ototoxicity was observed in the control group. Patients who were diagnosed with ototoxicity had no permanent audiometric threshold shifts in follow-up audiometric evaluation and these patients had no clinical complaints of difference in hearing.

**Conclusion:**

According to our results, audiometric threshold shifts can be detected in patients who undergo primary TKA using gentamicin loaded bone cement. However, no permanent shifts were observed during close follow-up.

## 1. Introduction

Cemented fixation is the most preferred method for total knee arthroplasty (TKA) among orthopedic surgeons, accepted as the gold standard according to our current knowledge [1,2]. Use of antibiotic-loaded bone cement (ALBC) in the primary TKA procedure has become a favorable option to prevent deep wound infection, which is a devastating complication after total joint arthroplasty [3]. Prophylactic systemic antibiotic administration may not be effective enough to prevent deep wound infection when used on its own. Therefore, ALBCs are used to achieve a local antimicrobial effect and their prophylactic use has been shown to be associated with lower risk of infection-based revision [4]. On the other hand, some concerns exist about routine use of ALBCs in TKA; these are the possibility of antibiotic resistance, allergic reaction, toxicity, impaired mechanical properties of bone cement, and increased costs [5,6]. 

Gentamicin is an aminoglycoside antibiotic, which provides local administration in the treatment of infection and has the advantages of high local concentrations, prolonged release, and low serum concentrations with its pharmacodynamic and pharmacokinetic properties [7,8]. Despite its serious complications such as nephrotoxicity and ototoxicity, gentamicin is a frequently used antimicrobial agent with a wide spectrum of activity and coverage of gram-negative bacteria [9,10]. In the literature, local gentamicin-induced ototoxicity was previously reported after using local gentamicin beads [11,12]. De Klaver et al. reported a 70-year-old patient who had ototoxicity after implantation of gentamicin beads [11]. Haydon et al. compared audiometric threshold shifts of systemic gentamicin treatment and local gentamicin bead treatment in osteomyelitis, reporting 28% versus 8% ototoxicity, respectively [12]. 

Gentamicin-loaded bone cement (GLBC) is often used in spacers when treating infected TKA; however, its routine use in primary TKA is still debated [13]. To date, no study could be found in the literature reporting the incidence of ototoxicity after using GLBC for primary TKA procedures. 

In our center, we have used GLBC in primary TKA procedures for years. We hypothesized that the use of GLBC in TKA may have a potential ototoxic effect, which could not be clinically diagnosed postoperatively. Therefore, the aim of the current study was to investigate the incidence of audiometric threshold shifts in patients who underwent TKA using GLBC in comparison with the ones who underwent TKA without GLBC.

## 2. Materials and methods

This prospective case-control study was performed after obtaining approval from the institutional ethical review board and was carried out in accordance with the Declaration of Helsinki. Informed consent was obtained from all patients. 

Seventy-five patients who underwent unilateral primary TKA participated consecutively in this study. Otoscopic examination and audiometric evaluation were performed for each patient preoperatively. Patients with normal middle ear and hearing status were included in the study. The exclusion criteria were: a) patients with medical history of renal failure or presently identified as having impaired renal function according to preoperative biochemical parameters, b) patients with medical history of otovestibular pathology, c) patients who had hearing loss according to preoperative audiometric evaluation, d) patients who were not able to undergo audiometric evaluation, e) patients who had ruptured eardrum, middle ear congestion, or infection according to preoperative otoscopic examination. 

Patients were grouped according to the type of polymethylmethacrylate (PMMA) bone cement used in the TKA procedure. The gentamicin group consisted of 40 patients who underwent primary TKA using GLBC, and the control group consisted of 29 patients who underwent primary TKA using standard PMMA bone cement without antibiotic. Patients’ age, sex, and American Society of Anesthesiologists (ASA) score were noted. All TKA procedures were performed by two surgeons under general anesthesia by the standard medial parapatellar approach. One surgeon used GLBC, which contained 0.5 g of gentamicin, and the other surgeon used standard bone cement. Both types of PMMA bone cement were prepared in the same manner by mixing liquid and powder contents (the first-generation cementing technique). All patients received postoperative intravenous cephalosporin, which was continued for 72 h, and low-molecular-weight heparin for thromboembolic prophylaxis until the end of the postoperative 4th week. 

Baseline pure-tone audiometric evaluation was performed before the TKA procedure for all patients by one audiometrist who was blinded to the type of bone cement used. A second audiometric evaluation was performed at the postoperative 3rd day. Control audiometric evaluation was performed weekly for patients who were diagnosed with ototoxicity. Test frequencies ranged from 500 Hz to 8000 Hz in octave intervals. Pure-tone thresholds were determined using a MADSEN Astera 2 clinical audiometer (Otometrics A/S,****Taastrup**, **Denmark). Postoperative audiometric threshold changes were evaluated and ototoxicity was diagnosed according to the criteria of the American Speech-Language-Hearing Association (ASHA), which define ototoxicity as ≥20 dB shift at one frequency or ≥10 dB shift at two adjacent frequencies [14]. 

### 2.1. Statistical analysis

Numeric variables were shown as mean ± standard deviation (SD) and categorical variables were defined as frequencies and percentiles. The comparison of the pre- and postoperative audiometric evaluation measurements within groups was performed by Mann–Whitney U test. Comparison of two independent groups was performed by Wilcoxon test for means and by Pearson chi-Square test for frequencies. Statistically, the significance level was defined as P < 0.05.

## 3. Results

**Figure 1:**
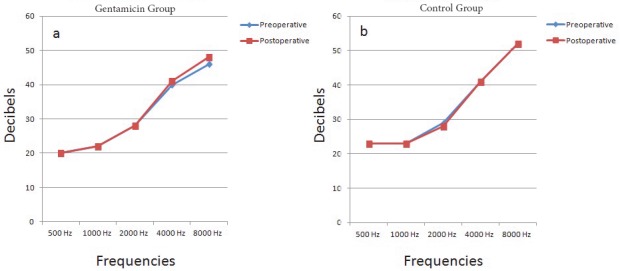
The line charts demonstrating the comparison of pre- and postoperative mean decibels at each frequency in (a) gentamicin group and (b) control group.

Patients’ demographics and comparison of groups are demonstrated in Table 1. Preoperative and postoperative audiometric evaluations of the two groups were compared for each frequency and no significant difference was obtained in the mean decibels between groups. In the gentamicin group, a significant difference was observed between preoperative and postoperative 3rd day audiometric evaluations in mean decibels at 8000 Hz (Table 2; Figures 1a and 1b). Ototoxicity was diagnosed according to postoperative audiometric threshold shifts in 8 of 40 patients (20%) in the gentamicin group, whereas no ototoxicity was observed in the control group. All patients who were diagnosed with ototoxicity had audiometric threshold shifts over 20 dB at 8000 Hz. The mean age of these patients was 66.2 ± 8.8 years (range: 56 to 85 years) and all patients were female. Postoperatively, none of the patients in either group had impaired renal function according to laboratory tests. During follow-up audiometric evaluations, patients who were diagnosed with ototoxicity had no permanent audiometric threshold shifts at a mean time of 4 weeks follow-up. These patients had no clinically evident deafness and noticed no hearing differences in daily living. 

**Table 1:** Comparison of demographics between groups.

	Gentamicin group(n = 40)	Control group(n = 29)	P-values
Sex			n.s.*
Female	34 (85%)	25 (86.2%)	
Male	6 (15%)	4 (13.8%)	
Age (years)	67.7 ± 8.3	67.3 ± 6.5	n.s.**
ASA score			n.s.*
1	0 (0%)	0 (0%)	
2	24 (60%)	8 (53.3%)	
3	15 (37.5%)	7 (46.7%)	
4	1 (2.5%)	0 (0%)	
n.s. = Statistically not significant.			
* P-value according to Pearson chi-Square test.			
** P-value according to t-test.			

**Table 2:** Comparison of audiometric evaluations between groups.

		Gentamicin group(n = 40)	Control group(n = 29)	P-values
500 Hz	Preop (dB)	20.1 ± 9.8	21 ± 9.6	n.s.**
	Postop (dB)	19.8 ± 8.8	20.6 ± 9.5	n.s.**
	Intragroup P-values	n.s.*	n.s.*	
1000 Hz	Preop (dB)	21.7 ± 10.2	21.5 ± 9.5	n.s.**
	Postop (dB)	22 ± 9.7	21.7 ± 9.3	n.s.**
	Intragroup P-values	n.s.*	n.s.*	
2000 Hz	Preop (dB)	27.7 ± 12.5	26.7 ± 11	n.s.**
	Postop (dB)	28.1 ± 12.3	26.5 ± 10.6	n.s.**
	Intragroup P-values	n.s.*	n.s.*	
4000 Hz	Preop (dB)	40.1 ± 18	39.6 ± 17.3	n.s.**
	Postop (dB)	41.2 ± 17.3	40.1 ± 17.3	n.s.**
	Intragroup P-values	n.s.*	n.s.*	
8000 Hz	Preop (dB)	46.1 ± 20.6	48.7 ± 21	n.s.**
	Postop (dB)	49.5 ± 21.1	48.7 ± 21.2	n.s.**
	Intragroup P-values	0.026*	n.s.*	
n.s. = Not statistically significant.				
* P-values according to Wilcoxon test.				
** P-values according to Mann–Whitney U test.				

## 4. Discussion

The aminoglycosides are among the most common ototoxic drugs causing cochlear and vestibular damage [15,16]. Ototoxicity of aminoglycosides may present with high-frequency deafness, which can only be diagnosed by audiometric evaluation, and its incidence has been reported up to 33% [17,18]. 

Gatell et al. reported that age was the most important predisposing factor for the development of ototoxicity in patients treated with aminoglycosides [19]. In our study, no significant difference was observed between the two groups in terms of age. In addition, those who were diagnosed with ototoxicity had a mean age of 66 years old, which was similar to study groups. 

Aminoglycoside doses, serum levels, and their relation with ototoxicity is another controversial issue in the literature [17]. In a review, Rao et al. indicated that a single dose is superior to multiple doses to avoid toxic levels; however, the authors reported no difference between groups in terms of nephrotoxicity and ototoxicity [20]. Ahmed et al. reviewed 103 patients over 23 years and reported that gentamicin can be vestibulotoxic at any dose, in any regimen, and at any serum level [21]. De Klaver et al. reported a sudden reversible hearing loss in a 70-year-old patient after administration of 400 mg of systemic gentamicin, with severe and irreversible hearing loss in the same patient after implantation of gentamicin-loaded beads [11]. In our study, GLBC containing 0.5 g of gentamicin was administered to patients in the gentamicin group and audiometric threshold shifts, which represent ototoxicity, occurred in 20% of our patients at the postoperative 3rd day. However, none of our patients had clinical complaints.

Pharmacodynamic studies showed that local administration of gentamicin produces low serum levels without toxic serum levels; however, several studies showed the development of ototoxicity and nephrotoxicity even after local gentamicin administration [10–12,22,23]. Downes et al. investigated the clearance of gentamicin in patients who underwent arthroplasty using GLBC [24]. The authors measured gentamicin levels in drainage fluid, serum, and urine, which decreased to negligible levels at the postoperative 5th day [24]. They reported two types of gentamicin release; one group showed a peak level within hours, while the other group showed a gradual peak between the postoperative 1st and 3rd days [24]. When we compared patients who underwent primary TKA using either GLBC or standard bone cement with no loaded antibiotics, we observed a reversible ototoxic effect of gentamicin according to the audiometric evaluation performed at the 3rd postoperative day. The detected ototoxicity was resolved gradually within a mean time of 4 weeks. The peak level within hours or gradual elevation of serum gentamicin level within the first few days after local administration of GLBC, which was demonstrated previously by Downes et al., may explain the mechanism of ototoxicity detected at the 3rd postoperative day in our patients [24]. Furthermore, in the case of systemic gentamicin administration as part of a postoperative antibiotic regimen, this mechanism may predispose to permanent audiometric threshold shifts in such patients. 

Two main limitations were noted in this study. First, serum levels of gentamicin were not investigated in this study to evaluate the toxic doses. However, it was previously reported that gentamicin could be ototoxic at any dose, in any regimen, and at any serum level [21]. Second, a relatively small number of patients were evaluated. However, this study was the first in the literature that reported audiometric threshold shifts diagnosed as ototoxicity after using gentamicin ALBC in primary TKA. Analyses of the data in comparison to a similar cohort as a control group provided a detailed understanding of gentamicin-related ototoxicity.

According to our results, audiometric threshold shifts can be detected in patients who undergo primary TKA using gentamicin-loaded bone cement. However, no permanent shifts were observed during close follow-up. Orthopedic surgeons should consider the potential risk of ototoxicity after using gentamicin-loaded bone cement in primary TKA even if 0.5 g of gentamicin is locally administrated.
